# Recurrent dengue outbreaks in Timor-Leste as an escalating public health emergency: An urgent call for action

**DOI:** 10.1016/j.nmni.2026.101879

**Published:** 2026-10-28

**Authors:** Kevin Smith P. Cabuhat, Christian Joseph N. Ong, Jerico B. Ogaya, Cipriano do Risario Pacheco, Rahmatullah Nazari, Don Eliseo Lucero-Prisno

**Affiliations:** a Basic Education Department, La Consolacion University Philippines, Malolos, Bulacan, Philippines; b Department of Biology, College of Science, De La Salle University, Manila, Philippines; c Research Services Office, Palompon Institute of Technology, Palompon, Leyte, Philippines; d Department of Medical Technology, Institute of Health Sciences and Nursing, Far Eastern University, Manila, Philippines; e Department of Nutrition Science, Faculty of Public Health, Univercidade da Paz, Dili, Democratic Republic of Timor-Leste; f Medical Research Center, Asas Medical University, Kabul, Afghanistan; g Department of Global Health and Development, London School of Hygiene and Tropical Medicine, London, United Kingdom; h Research and Innovation Office, Southern Leyte State University, Sogod, Southern Leyte, Philippines; i Research and Development and Community Extension, John B. Lacson Maritime University, Iloilo, Philippines

**Keywords:** Dengue, Timor-Leste, Disease outbreak, Public health emergency, Surveillance

Dear Editor,

Timor-Leste is currently experiencing a severe dengue outbreak. As of 6 February 2026, Dili has reported over one thousand confirmed cases and five pediatric deaths. Hospitals have exceeded bed capacity, with patients receiving care in chairs in overcrowded wards. Essential medical supplies, including intravenous fluids, laboratory reagents and basic monitoring devices are in acute shortage [1]. This is the third major dengue outbreak in Dili since 2020 [2], [3].

Recurrent outbreaks in a single setting indicate recurring health system strain, not isolated events. Dengue management is well-established: early diagnosis, monitoring, and supportive care substantially reduce mortality. During recent outbreaks, hospital capacity was exceeded and essential supplies were in shortage [1], [3], with persistent diagnostic and laboratory constraints [2], [3], suggesting deficits in surge capacity, supply-chain resilience, and surveillance infrastructure.

In the 2022 outbreak, Dili recorded 3,904 dengue cases and 35 deaths (case fatality rate [CFR] 0.9%) [3]. Preliminary data from the current 2026 outbreak indicate approximately 1,000 cases with 5 pediatric deaths (CFR 0.5%) [1]. While the current CFR is lower, it remains clinically significant, and the continued occurrence of pediatric deaths is concerning. Direct comparison is limited by denominator underreporting during surge periods.

Timor-Leste has made notable gains in laboratory capacity in recent years, including expanded serological and molecular diagnostic capabilities at the National Health Laboratory. However, during outbreak surges, molecular testing remains centralized in a single facility in Dili, creating diagnostic bottlenecks. Routine serotype surveillance is not conducted between outbreaks, impeding early warning of severe disease risk. Additionally, the Ministry of Health has recently initiated a Wolbachia-infected mosquito deployment program in Dili, which represents a promising integrated vector control strategy. This program is in its early stages and does not yet replace the need for emergency surge capacity, supply chain resilience, or sustained surveillance.

Globally, dengue incidence is increasing, and small island states in Southeast Asia and the Pacific are disproportionately vulnerable [3]. For Timor-Leste, the interval between major outbreaks has shortened, and health system strain has accumulated.

We propose three specific, actionable measures. These recommendations are based on public surveillance reports and preliminary outbreak data, not primary data from the authors, and are presented as policy proposals rather than empirical conclusions. First, the Ministry of Health should request technical deployment through the ASEAN Coordinating Centre for Humanitarian Assistance on disaster management (AHA Centre) [4]. The ASEAN regional mechanism for disaster health management explicitly includes response to public health emergencies, including disease outbreaks, and enables rapid deployment of clinical logistics and emergency medical teams**.** Second, we propose that WHO consider treating Timor-Leste’s recurrent dengue outbreaks as a single protracted emergency rather than discrete events. This policy proposal aims to highlight cumulative health-system strain and is not based on an empirical analysis of WHO grading criteria. WHO defines protracted emergencies as those persisting for more than six months. Current grading criteria, however, do not formally account for cumulative health system strain from recurrent seasonal outbreaks, which obscures the need for multi-year surge support. Third, development partners should prioritize establishing sustainable serotype surveillance in Dili and Baucau. Without knowledge of circulating serotypes, the country cannot anticipate severe dengue risk or prepare for future vaccine introduction [5] (see Table 1).

**Table 1:** Summary of current gaps and proposed measures.

Current Gap	Proposed Measure
Lack of surge clinical logistics and emergency medical team capacity	Request technical deployment through the AHA Centre
Recurrent outbreaks classified as discrete events, obscuring cumulative health system strain	WHO to consider reclassifying recurrent dengue outbreaks as a single protracted emergency
No routine serotype surveillance between outbreaks	Establish sustainable serotype surveillance in Dili and Baucau
Centralized molecular testing creating diagnostic bottlenecks during surges	Decentralize molecular testing capacity and strengthen laboratory network

These interventions are feasible, aligned with existing regional frameworks, and do not require new institutions or treaties. What is required is recognition that Timor-Leste’s dengue crisis is not seasonal but structural. Beyond publication, the authors commit to submitting these recommendations formally to the Timor-Leste Ministry of Health, WHO Country Office, and the AHA Centre. Without sustained, pre-positioned response capacity, the next outbreak may follow the next rains.

## CRediT authorship contribution statement

**Kevin Smith P. Cabuhat:** Writing – review & editing, Writing – original draft, Resources, Formal analysis, Data curation, Conceptualization. **Christian Joseph N. Ong:** Writing – review & editing, Writing – original draft, Project administration, Investigation, Data curation, Conceptualization. **Jerico B. Ogaya:** Writing – review & editing, Writing – original draft, Investigation. **Cipriano do Risario Pacheco:** Writing – review & editing, Writing – original draft, Investigation. **Rahmatullah Nazari:** Writing – review & editing, Writing – original draft, Validation, Data curation. **Don Eliseo Lucero-Prisno:** Writing – review & editing, Validation, Supervision.

## Data availability

No new data were created or analyzed in this study. All data referenced are derived from publicly available sources cited within the article.

## Ethical approval

Not required for this commentary/letter.

## Declaration of generative AI and AI-assisted technologies

During the preparation of this work, the authors used Paperpal AI (https://paperpal.com/) to improve language clarity and readability. After using this tool, the authors reviewed and edited the content as needed and take full responsibility for the content of the published article.

## Funding

This research did not receive any specific grant from funding agencies in the public, commercial, or not-for-profit sectors.

## Declaration of competing interest

The authors declare that they have no known competing financial interests or personal relationships that could have appeared to influence the work reported in this paper.
